# Diet Recommendations for Hospitalized Patients With Inflammatory Bowel Disease: Better Options Than Nil Per Os

**DOI:** 10.1093/crocol/otaa059

**Published:** 2020-07-17

**Authors:** Sonali Palchaudhuri, Lindsey Albenberg, James D Lewis

**Affiliations:** 1 Division of Gastroenterology and Hepatology, Perelman School of Medicine at the University of Pennsylvania, Philadelphia, Pennsylvania, USA; 2 Division of Pediatric Gastroenterology, Hepatology, and Nutrition, The Children’s Hospital of Philadelphia, Philadelphia, Pennsylvania, USA

**Keywords:** nutrition, inflammatory bowel disease, inpatient, diet, hospitalization, dietary interventions, therapeutic diets, Crohn disease, ulcerative colitis, flare food

## Abstract

Hospitalizations are a time when providers often have uncertainty about what to feed patients with inflammatory bowel disease (IBD). While there are many trials evaluating the role of diet in the management of IBD, the role of diet for the hospitalized patient is less clear. The hospitalization may serve as an opportunity to educate patients about the role of diet, try different diets, and develop dietary recommendations for after discharge. Here, we review the literature for dietary considerations during hospitalizations and acute settings, as well as upon discharge. Patients with IBD benefit from screening and nutritional support for malnutrition and nutritional deficiencies. Enteral nutrition and exclusion diets are promising as induction and maintenance therapies, but no specific recommendations during hospitalization for adult patients are available currently. There are very few reasons to enforce bowel rest or clear liquids other than bowel obstruction, uncontrolled sepsis, or need for urgent or emergent surgery; most patients—including many with penetrating or stricturing disease—benefit from feeding in whichever capacity is tolerated, with enteral and parenteral nutrition used as needed to reach nutritional goals. Future studies are needed to define how the use of different diets can influence the outcomes of patients hospitalized for IBD.

## INTRODUCTION

Many studies have concluded that diet plays a role in the development of inflammatory bowel disease (IBD) through a proinflammatory effect.^1^ Dietary components associated with an increased risk include animal protein, heme iron, sulfur, refined sugars, and high trans-fat, while components associated with a decreased risk are fiber, fruit, vegetables, and high omega-3 fatty acids.^2^ There have been many trials looking into the role of diet in management of IBD. Defined formula diets have been included in some guidelines as induction or adjunctive therapy for Crohn disease (CD), with no specific recommendation for ulcerative colitis (UC).^3^

**Figure 1.**
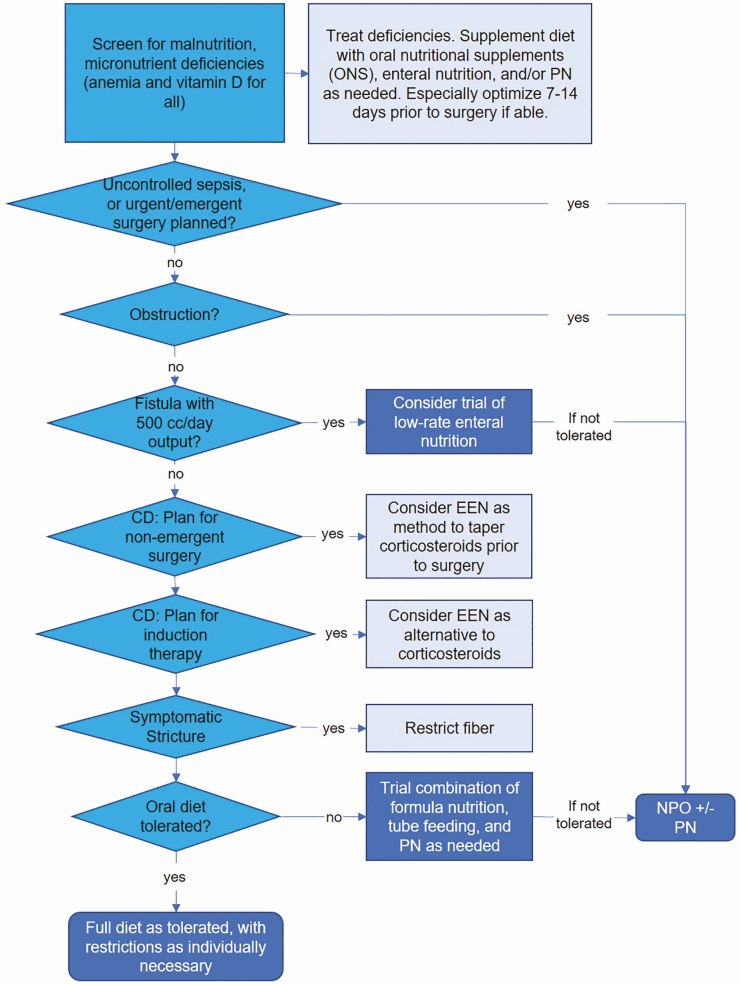
Pathway for nutrition and diet recommendations. The darkest blue boxes indicate endpoints while the lightest boxes indicate adjunctive measures.

The role of diet in management of IBD for the hospitalized patient is particularly under studied, with most guidelines making no specific recommendations.^3–5^ Historically, the accepted diet for patients with CD was to avoid “roughage,” with no other suggestions or stipulations.^6^ A low-residue diet is still widely used, with periods of no oral diet and only clear liquids also frequently employed in hospitalized patients. This review article offers guidance for nutritional recommendations during hospitalization, when providers may have the most concern about the risks of feeding. The hospitalization may also serve as an opportunity to educate patients about diet, try different diets and develop dietary habits for after discharge. Here, we review the literature for dietary considerations during hospitalizations and acute settings and offer a general framework for nutritional recommendations for patients with IBD (Fig. 1).

## THE ACUTE CARE SETTING AS AN OPPORTUNITY TO SCREEN FOR NUTRITIONAL DEFICIENCIES

A hospitalization—with easy access to laboratory evaluations and therapies—is an opportune time to evaluate for and correct nutritional deficiencies. It is important to note that plasma measurements of micronutrients may be affected by active inflammation due to being bound to acute-phase reactants like albumin, and as such assessments are more reliable during remission.^7^ Still, a thoughtful interpretation of laboratory tests combined with patient history and clinical assessment may lead to early diagnosis and treatment of common nutritional comorbidities.

### Malnutrition in Patients With IBD

In patients with IBD, malnutrition can occur due to a confluence of mechanisms: decreased oral intake due to fear of symptoms and/or self-imposed diet restrictions, enteral protein loss, increased requirements secondary to active inflammation, malabsorption, and drug interference.^8^ Thus, malnutrition is relatively common in patients with IBD; small studies defining malnutrition with varying methods estimate the prevalence of malnutrition is 25%–70%.^9^ Patients with malnutrition are more likely to be hospitalized from the emergency room [odds ratio (OR) 6.3, 95% confidence interval (CI) 5.8–6.8].^10^

Malnutrition is especially common among those hospitalized. In a national database study evaluating the prevalence of the specific discharge codes for protein-energy malnutrition, patients with CD or UC both had a greater prevalence of malnutrition than non-IBD patients (6.1% and 7.2% vs 1.8%).^11^ While this is likely an underestimate of the prevalence due to the study design, it shows that malnutrition is more common among hospitalized patients with IBD than other hospitalized patients.

Studies from large nationwide databases of hospitalized patients with IBD identified that malnutrition was associated with increased in-hospital mortality, length of stay, and total charges,^11^ and patients hospitalized with CD were more likely to get bowel surgery or prolonged hospitalization stay (OR 3.7 of having either, 95% CI 3.2–4.2).^12^ Multiple studies show that malnutrition is an independent risk factor for hospital complications: venous thromboembolism, nonelective surgery, longer admission, and increased mortality.^4^ Citing the prevalence and predictive value of malnutrition, guidelines from the European Society for Clinical Nutrition and Metabolism (ESPEN) and British Society of Gastroenterology (BSG) recommend screening all patients with IBD at the time of diagnosis and regularly afterwards^4,5^ while the American College of Gastroenterology (ACG) guidelines recommend screening early in patients with CD.^13^ These guidelines are not specific to hospitalized patients, but the hospital is often the place of diagnosis and also offers an opportunity for screening for those with established diagnoses.

It is important to recognize that patients without a low body mass index (BMI) may still be malnourished and warrant screening. Sarcopenia, characterized by the loss of skeletal muscle mass and function,^14^ was found to be present in 60% of patients with CD in clinical remission, in whom the mean BMI was 20 kg/m^2^ (within normal range).^15^ In a retrospective analysis of all patients with IBD started on a tumor necrosis factor inhibitor (anti-TNF), Adams et al showed that sarcopenia defined by low muscle mass on imaging was present in 20% of the overweight patients.^16^ They also found these patients to be more likely to require surgery than nonsarcopenic counterparts^16^ while Bamba et al showed that sarcopenia, based on imaging was shown to be an independent prognostic factor for intestinal resection in patients with IBD.^17^

Screening and treating malnutrition during hospitalizations can impact outcomes. In a randomized controlled trial of all medicine patients at nutritional risk, which included patients with IBD, the multicenter Swiss EFFORT trial found that individualized nutritional support during hospitalization decreased adverse outcomes within 30 days and all-cause mortality when compared to standard care.^18^

There are multiple scoring methods for malnutrition, involving screening, diagnosis, and monitoring. The Malnutrition Universal Screening Tool (MUST)^19^ is easy to implement: the score is calculated based on BMI, percent of weight loss, and whether there has been no nutritional intake for >5 days. MUST can be used for weekly screening and monitoring in the hospital for all patients. Other methods for screening include the Malnutrition Screening Tool (MST), Mini Nutritional Assessment-Short Form (MNA-SF) for older adults only, the Nutritional Risk Screening (NRS-202), and the Simplified Nutritional Assessment Questionnaire (SNAQ).^20^ The Global Leadership Initiative on Malnutrition (GLIM) developed a consensus document for the diagnosis and severity grading of malnutrition, based on criteria from MUST and other existing approaches. The GLIM criteria for diagnosis are to meet at least 1 phenotypic criterion (nonvolitional weight loss, low BMI, and reduced muscle mass) and 1 etiologic criterion (reduced food intake or assimilation, and inflammation or disease burden), where grading is dependent on the degree of abnormality of the phenotypic criteria.^21^

Understanding that the protein requirements are increased during active IBD, guidelines suggest increased protein intake (to 1.2–1.5 g/kg/d in adults).^4^ Management of malnutrition is best guided by a nutrition support team, who can recommend oral supplements, enteral nutrition, and parenteral nutrition (PN) based on nutritional needs, available resources, and patient goal/preferences.

### Specific Vitamin and Mineral Deficiencies

Anemia is highly prevalent in patients with IBD. A European meta-analysis of studies from 2007 to 2012 reported a prevalence of 27% in patients with CD and 21% in patients with UC, where 57% of anemic patients were iron deficient.^22^ Both CD and UC can cause iron deficiency via intestinal blood loss through ulcerated mucosal surface and decreased nutritional intake, and patients with CD may have malabsorption if there is disease involvement in the duodenum and upper jejunum.^23,24^ Studies from the 1960s and 1970s report the prevalence of anemia in hospitalized patients with CD to be as high as 73%; while the rates may be lower now, it is generally accepted that the prevalence of anemia is higher in hospitalized patients with acute disease.^25^ Complicating the evaluation of iron deficiency anemia is that acute inflammation may increase positive acute-phase proteins including ferritin. The Crohn’s & Colitis Foundation’s pathway based on expert consensus for anemia in patients with IBD provides 2 definitions for iron store depletion in the context of inflammation (ferritin <100 ng/mL, or ferritin ≥100 ng/mL and transferrin saturation <20%).^26^

Iron supplementation for iron deficiency anemia is recommended, and the consensus is to use intravenous iron as first-line treatment in patients with clinically active IBD. This is due to concerns regarding oral iron absorption in patients with CD and overall poor tolerance with oral iron.^4,24,26^ Providing intravenous iron during a hospitalization avoids the practical difficulties of waiting to treat after discharge.

The second most common cause of anemia in the IBD population is thought to be anemia of chronic disease, though vague diagnostic criteria make it difficult to assess a true prevalence.^25,27^ While B12 deficiency is listed as cause to consider,^23,26^ a systematic review of 42 articles by Battat et al concluded that CD without ileal resection, even with ileal involvement, and UC do not increase the risk for B12 deficiency; only ileal resections greater than 30 cm were associated with B12 deficiency in CD.^28^ Furthermore, of patients with IBD and anemia, B12 deficiency is attributed as the cause in very few cases. Folate deficiency anemia is also less common,^27^ especially with the less frequent use of sulfasalazine.

Patients with IBD are at increased risk for low bone mineral density and osteoporosis.^29^ This is thought to be due to a combination of multiple factors: chronic malabsorption and diet changes leading to calcium and vitamin D deficiencies, disruption in bone formation and regeneration due to inflammatory cytokines, and corticosteroid use causing osteoblast apoptosis.^30,31^ A study of patients with UC showed the prevalence of vitamin D deficiency was 55%, and that vitamin D deficiency was significantly correlated with active colitis as well as with need for steroids^32^; vitamin D deficiency and steroid use are both risk factors for low bone mineral density.^33^ Lower vitamin D levels were correlated with higher rates of *Clostridium difficile* infection^34^ and earlier cessation of anti-TNF alpha therapy.^35^ Other studies have suggested that normalizing vitamin D levels decrease risk of surgery,^36^ reduce healthcare utilization,^37^ and reduce the relapse rate at 3 months and 1 year.^38^

Multiple studies have shown that vitamin D can influence the gut microbiome and intestinal immune responses in both human and animal models,^39,40^ so it is still not clear if the relationships described between improved vitamin D and clinical outcomes is correlative or causative. However, since vitamin D supplementation is otherwise appropriate, and may be helpful in the management of acute IBD with low risk of harm, it is reasonable to evaluate for vitamin D deficiency and initiate management while hospitalized for acute colitis or upon discharge.

Other deficiencies can include vitamin B6 and zinc.^41,42^ Low vitamin B6 levels in patients with IBD have been associated with hyperhomocysteinemia,^42^ where both low vitamin B6 levels and hyperhomocysteinemia have long been shown to be risk factors for both arterial and venous thrombosis.^43,44^ This is of specific concern in the IBD population, known to be at higher risk for thrombotic events.^45,46^ Zinc, absorbed in the small intestine, is a cofactor involved in immune function and tissue repair. Among patients with IBD, zinc deficiency has been associated with increased risk of hospitalizations, surgeries, and disease-related complications including fistulas and strictures, with a suggestion of improvement in these outcomes with normalization of zinc.^47^ As with other conditions leading to chronic diarrhea or malabsorptive disorders, it is reasonable during a hospitalization to assess the level of disease activity, evaluate for specific nutritional deficiencies that may arise, and respond with correction of specific deficiencies as well as possible overlapping overall poor nutrition.

## DIET AS PRIMARY THERAPY

### Role of Nutrition for Inducing Remission

Exclusive enteral nutrition (EEN) with an elemental or polymeric diet has been demonstrated to induce clinical and endoscopic remission in CD. Being amino acid based, an elemental diet is designed to be completely absorbed in the duodenum and proximal jejunum, thus providing bowel rest for the distal small bowel and colon. In theory, the composition of most low fat elemental diets allows for absorption with minimal pancreatic enzyme action and biliary stimulation.^48^ In contrast to elemental diets, polymeric diets include whole proteins but a 2018 Cochrane review demonstrated that there is no difference in efficacy for induction of CD remission between elemental and polymeric formulas or between low and high fat.^49^

It is hypothesized that EEN promotes mucosal healing in the gastrointestinal tract by changing the intestinal microbiome,^50^ as well as by “reducing intestinal permeability, enhancing barrier defense and adaptation, and promoting a reduction of pro-inflammatory cytokines.^4^” However, the changes in the gut microbiome with EEN result in a more rather than less “dysbiotic” composition until the inflammation is resolved.^51^ Thus, other mechanisms may be mediating the therapeutic benefit.

Several studies have demonstrated that EEN can induce remission among children with CD, including studies where EEN has been directly compared to corticosteroids.^52^ Accordingly, the ESPGHAN/NASPGHAN guidelines state that EEN has the same efficacy as oral steroids in the induction of remission of children with active luminal CD and should be considered as first-line therapy to induce remission in this population.^53^

Regarding adults, the data and guidelines are less clear. Okada et al conducted a controlled trial on 20 adult patients with active CD comparing EEN to steroids, where they defined complete remission as improvement in clinical symptoms, nutritional state, inflammatory parameters, and radiographic bowel lesions. At 6 weeks, they found that EEN led to complete remission in 8 of 10 patients vs 1 of 9 evaluated patients on steroids.^54^ Citing this study and prior observational studies, practice guidelines in Japan suggest an EEN as a primary option for induction therapy for adult patients with CD.^55^ While follow-up studies in Japan use EEN as a standard induction therapy,^56^ EEN is infrequently used as induction therapy for CD in the USA and Europe.

The 2018 Cochrane review comparing EEN to corticosteroid therapy to induce clinical remission in adults with active CD concluded that the data are of very low quality and in favor of corticosteroid therapy. The authors note that patients on EEN were more likely to withdraw due to intolerance.^49^ Many of the studies were used elemental diets despite polymeric formulas being more palatable and generally preferred.

The British Society of Gastroenterology acknowledges the low-quality evidence and suggests EEN for induction therapy in patients with mild-to-moderate CD who are interested to avoid corticosteroids and will adhere strictly to EEN for up to 8 weeks.^5^ Symptomatic improvement with EEN may take 10 days or longer, but many patients will respond sooner. The ACG does not comment on the use of EEN for induction therapy in their guidelines for the management of CD in adults,^13^ nor UC in adults.^57^

For patients with stricturing or penetrating disease, there are less data on the use of EEN. A 10-year retrospective review by Teahon et al in 1990 included 35 patients with strictures and found there was a short-term remission in 33 patients (94%); of the 21 patients who resumed a normal diet, 13 maintained remission for a mean of 29 months. Among 10 patients with fistulas: 8 achieved remission and all 4 who resumed normal diet relapsed within 1 month.^58^ This study was done prior to availability of anti-TNF therapies. Encouragingly, a 2017 retrospective study of patients with stricturing or penetrating CD designed to evaluate EEN’s impact on postoperative complications found that 25% of patients started on EEN had clinical improvement such that they no longer required surgery.^59^ This suggests that EEN can be used as a bridge strategy to surgery, with some possibility it may alleviate the need for surgery.

### Exclusion Diets/Therapeutic Whole Food Diets

Given the limitations of EEN, diets which allow more flexibility are increasingly being studied. In children, a multicenter observational cohort study found that both EEN and anti-TNF therapy improved symptoms and intestinal inflammation markers better than partial enteral nutrition (PEN).^60^ A more recent randomized control trial in children with mild-to-moderate CD demonstrated that a Crohn’s Disease Exclusion Diet (CDED) with PEN was comparable to EEN in inducing remission by week 6. Furthermore, CDED with PEN was better tolerated than EEN, and superior in sustaining remission by week 12 after reintroducing foods to the EEN group.^61^ This trial supported its use as first-line over EEN. More trials aiming to improve tolerance with PEN are under way.

Published guidelines generally do not directly address the role of PEN, EEN, or therapeutic whole food diets in hospitalized patients. However, it may be appropriate to initiate PEN, EEN, or a therapeutic whole food diet in the setting of an acute flare as a component of both induction therapy and long-term maintenance therapy. From a practical perspective, starting EEN as an inpatient may offer access to resources for calculating calorie needs, teaching patients and families how to administer the therapy, and obtain insurance approval, as well as monitoring for tolerance and adjusting as needed.

## DIET AS ADJUNCTIVE THERAPY

### Enteral Nutrition and Anti-TNFa

Initial response to anti-TNF therapy is inadequate and loss of response remains an issue.^62,63^ Observational studies in Japan, where enteral nutrition is widely used as maintenance therapy for CD, have showed that concomitant use of enteral nutrition was associated with sustained response to infliximab.^64,65^ In a meta-analysis of 4 Japanese studies evaluating patients with CD, the use of combination therapy of infliximab with specialized enteral nutrition resulted in higher odds of maintaining clinical remission compared to infliximab alone (OR 2.7, CI 1.7–4.3), with a number needed to treat of 4.^66^ This suggests that starting enteral nutrition with infliximab, and continuing on discharge, may be advantageous.

### Perioperative Nutrition

Nutrition is a component of Enhanced Recovery Pathways (ERP), also known as Enhanced Recovery After Surgery (ERAS). Surgical nutrition guidelines strongly support preoperative nutrition to improve postoperative outcomes as part of ERAS for all patients undergoing abdominal surgery, using enteral nutrition first and then PN if necessary.^67^

In a 10-year retrospective cohort study, patients with malnutrition undergoing IBD surgery who received preoperative PN compared to those who did not had a similar incidence of noninfectious complications, despite having a higher proportion of severe malnutrition and severe disease, and greater than 60 days of PN resulted in a reduction in noninfectious postoperative complications with no associated increase in infectious complications.^68^ A 2017 systematic review of 14 original studies and 15 reviews concluded that the data are heterogeneous but consistently found that preoperative malnutrition for patients with IBD was a risk factor for postoperative complications, and nutritional support—both enteral and parenteral—decreased postoperative morbidity.^69^ Therefore, the consensus is to start enteral nutrition if a patient with IBD undergoing surgery is not able to meet 60% of nutritional needs.^70^ ESPEN guidelines encourage delaying nonemergent surgical intervention for patients with IBD and malnutrition by 7–14 days so to initiate nutrition support before surgery.^4^

A retrospective review of 161 consecutive patients with CD undergoing ileocecal resection found that poor nutritional status, defined by greater than 10% weight loss, was 1 of 4 independent factors that increased the risk of postoperative intra-abdominal septic complications, with an OR of by 6.2 (95% CI 1.8–22.5).^71^ In a meta-analysis of 5 studies of patients with CD, nutrition support decreased the rate of postoperative complications from 60% to 20%, with a number needed to treat of 2.^72^ Furthermore, in patients given EEN prior to surgery had a reduced length of surgery as well as a 9-fold decrease in postoperative abscess and/or anastomotic leak compared to matched controls without preoperative EEN.^59^

Fewer studies focus on patients with UC.^73^ Despite clear evidence, expert opinion is to optimize nutrition in patients with UC prior to surgery.^7^ A retrospective review of 235 patients with UC undergoing surgery compared those initiated on PN vs those without and concluded that there was no difference in postoperative complications when line infections were excluded, despite the group with pre-op PN being generally more ill^74^; this study does not support routine preoperative PN due to the risk of line infection, and suggests patient selection is key.

For patients with complicated IBD who benefit from longer-term pre-op PN for nutritional support or require bowel rest for obstructions, a small study found that PN at home instead of prolonged hospitalization or early surgery resulted in fewer high-risk early surgeries, and improved quality of life while lowering costs.^75^

Another threat to malnourished patients is that an acute depletion of arginine from surgical stress can impair T-cell function and wound healing, but this is modifiable with supplementation perioperatively.^76^ Therefore, it is recommended to provide perioperative and postoperative immune modulating nutrients (arginine, omega-3 fatty acids, and ribonucleotides, either as supplements or as included in feeds) to malnourished patients undergoing gastrointestinal surgery to reduce infective complications and shorten length of stay.^67^

With regard to nutrition after surgery, surgical ERAS guidelines suggest resuming normal oral diet as soon as 4 hours after surgery in patients with a new nondiverted colorectal anastomosis to decrease rates of infectious complications and avoid delayed recovery.^67^ Liska et al conducted a prospective study evaluating the outcomes of implementing an ERAS protocol in patients with IBD, where one of the components was early initiation of solid diet.^77^ The percentage of patients with solid diet on postoperative day 1 increased from 10% to 76% with implementation of the protocol; bundled with other parts of the protocol, implementation resulted in decreased length of stay and hospital costs without increasing the risk of postoperative complications.

## FOOD RESTRICTIONS

### Who Is Kept NPO and Who Needs to Be NPO?

A retrospective review of all patients admitted to hospital medicine services showed that half experienced a period of fasting and 25% of orders for *nil per os* (NPO) were deemed unnecessary.^78^ Specifically for IBD, 76% of 187 UC-related admissions to the medicine service at a Canadian tertiary care IBD referral center included an order for NPO or clear liquid diet, and a review suggested that 44% of orders were unjustified.^79^ In this study, the authors commented that more of the inappropriate orders were by a general medicine team awaiting consultation with the gastroenterology team, suggesting that many orders for NPO or clear liquid diet may be out of caution when the plan for endoscopy in particular is not yet known. This concern is to be balanced with the concern that time spent as NPO or on a clear liquid diet increases the risk for malnutrition.

### The Role of PN

In 1986, a European two-center randomized controlled trial on 47 hospitalized patients with active colitis from IBD necessitating IV corticosteroid therapy compared bowel rest with PN to oral diet. They found no benefit with PN and a nonsignificant trend toward higher need for surgical treatment in patients with UC.^80^ This echoed the results of a 1980 trial of 36 patients with IBD on corticosteroids, where total bowel rest with intravenous support did not have a therapeutic effect on acute colitis.^81^ Two small randomized controlled studies concluded that PN and EEN were equally effective in inducing clinical remission in CD,^82,83^ while another randomized trial on patients with active UC found a polymeric diet and PN to be comparable as adjunctive nutritional therapy to corticosteroids.^84^ It is widely accepted now that NPO by itself is not therapeutic for acute UC and bowel rest with PN) is not recommended as primary therapy of IBD unless bowel rest or PN is otherwise indicated.^57,85,86^

Multiple studies concluded that PN was associated with more frequent abnormalities in liver enzymes than EEN and did not have a better rate of inducing remission in patients with active CD, while EN is safer, cheaper, and nutritionally effective.^87^ Thus, for patients with UC, regular food is recommended and for patients with CD, EN is preferable to PN when possible.

Patients with IBD may fit one of several scenarios where bowel rest is necessary. Indications for using PN are when oral or tube feeding is not possible due to a dysfunctional or short gastrointestinal (GI) tract, when there is an obstructed bowel with no possibility of placing a postobstruction feeding tube, or when there are complications like an anastomotic leak or a high output intestinal fistula.^4,86^

The other use of PN is to supplement oral diet without bowel rest. A meta-analysis of 10 trials among 164 individuals with either CD or UC who received PN treatment in combination with food suggested there was improvement in disease activity and serum albumin measurements, with greater effect with more time.^87^ Nonetheless, this should be reserved for those who are unable to achieve sufficient nutrition with regular food and/or nutritional supplements.

### Management of Patients With Abscesses and Fistulas

There are no randomized controlled trials or prospective observational data on the type of feeding or the benefits of bowel rest in patients with abscesses or fistulas. Often patients are given PN in order to allow bowel rest, but there is scant literature to support this practice. The first step of management should always be to control infections. Once an abscess has been drained and it is established that there is no free perforation or need for emergent surgery, choice of nutrition is paramount. While PN and bowel rest have been shown to result in high rates of spontaneous closure of enterocutaneous fistulas (ECFs), it has not been proven to reduce overall mortality in patients with an ECF, possibly due to septic complications.^88^ Management of central lines and the rate of line infections have appreciably changed since many of these studies were conducted, so the risk to benefit balance of PN for patients with ECF may be more favorable today. Nonetheless, many patients may be able to tolerate oral nutrition following drainage of all abscess collections. Patients with high output ECFs (ie, >500 cc/d) may need PN for both nutrition and to help maintain fluid and electrolyte balance.

An elemental diet is an alternative to PN for some patients with high output ECF. In 1974, Rocchio et al reported that 29 of 35 patients with high output fistulas had significant decrease in fistula drainage with the slow infusion of an elemental diet. Of the 10 patients with IBD, spontaneous closure occurred in 7 patients, all of whom had mid or low fistulas.^48^ Offering nutritional support to correct malnourishment and reduce risk of sepsis is essential to reduce ECF morbidity and mortality, and recommendations are to use enteral nutrition instead of PN when possible.^88^

### Fiber and Low-Residue Diets in Patients With and Without Strictures

While a low-fiber diet is often ordered during hospitalization, there are limited data studying if this is beneficial. There are weak recommendations to reduce high-fiber foods during symptomatic flares for both CD and UC.^89,90^ Prior guidelines from multiple dietary and gastroenterologist groups like the Academy of Nutrition and Dietetics and ACG recommend against high-fiber foods during CD flares or in the presence of fistulas and strictures.^13,89^ ESPEN specifically recommends that patients with CD with intestinal strictures or stenosis in combination with obstructive symptoms adapt the texture of their diet, or use poststenosis enteral nutrition, though the guideline acknowledges there are no robust data to support this suggestion.^4^ Despite the lack of strong evidence from clinical trials, anecdotally, many patients note that high-fiber foods worsen their symptoms^91^ and as such it may be reasonable to use a low-fiber diet in the early phase of a severe flare of IBD if higher fiber foods worsen symptoms. The diet can be liberalized to include more fiber as the patient improves.

For hospitalized patients with symptomatic strictures, it is logical to avoid high-fiber foods that may cause mechanical obstruction, though there are no data to define the level of stricturing or amount of fiber.

### Other Food Restrictions

It is common to offer restricted diets to hospitalized patients, though there are no sufficient data to recommend this for all patients, even during an acute flare. The following discussion on restrictions are relevant for patients at any stage of care.

Dairy is a common food category in many elimination diets studied^92^ and was listed as a food to avoid in prior guidelines.^93^ A 2016 review of lactose digestion and dairy food effects in patients with IBD concluded that the small bowel involvement of CD can produce secondary lactose maldigestion, but it is still unclear if disease activity causes lactose maldigestion.^94^ It is now accepted that there is a higher prevalence of irritable bowel syndrome among patients with IBD,^94,95^ which may account for symptoms of food intolerances like lactose intolerance. Conversely, dairy restriction may have negative impacts, with bone heath, weight gain, and colorectal cancer risk reduction observed as benefits from dairy foods.^94^ Accordingly, dairy products could be consumed as tolerated, although authors of a guidance document from the International Organization for the Study of Inflammatory Bowel Disease were unable to come to a consensus on the appropriateness of consuming dairy products.^96^

Cohen et al surveyed 6768 patients with IBD to identify foods patients believed correlated with symptoms. They found that yogurt and rice were most frequently reported to improve symptoms in all IBD disease categories and patients with UC and a pouch also reported bananas as helpful.^91^ Conversely, the foods that patients in most disease categories reported to worsen symptoms included: nonleafy and leafy vegetables, spicy foods, fruit, nuts, fried foods, milk, red meat, soda, popcorn, dairy, alcohol, high-fiber foods, corn, fatty foods, seeds, coffee, and beans. This diverse group of foods are also among those known to cause GI distress for patients without IBD, so it may be that these foods are causing disease-unrelated symptoms. Without further evidence, it seems prudent to ask hospitalized patients to only avoid foods that they have found to be personally exacerbating, and only provide these lists of foods as suggestions for which foods to trial exclusion.

## PLANS AFTER DISCHARGE

There have been many diets proposed to keep patients with IBD in remission, that involve either the addition of foods, exclusion of food categories, or replacement with nutritional formulas like EEN or PEN.^97^ Most studies have had small sample sizes and no “therapeutic diets” have received endorsement in society guidelines.

In a Japanese randomized controlled trial of 51 patients recently induced into remission, mostly through EEN or PN during hospitalization, PEN was associated with reduced relapse rate compared to free, unrestricted diet.^56^ While there was no comparison to other medications for maintenance therapy, and some received azathioprine prior and during the study, they concluded that PEN was a tolerable and reasonable maintenance therapy option in patients intolerant or resistant to immunosuppressive agents.

Given the prevalence of IBS in patients with IBD, a low-FODMAP diet (exclusion of fermentable oligo-, di-, and mono-saccharides and polyols) has been studied and found to help some patients control symptoms.^92^ This may be most useful in those with persistent symptoms despite resolution of detectable inflammation. A recent clinical trial of patients with persistent symptoms despite healing of inflammation showed that the low-FODMAP diet was superior to a free diet to improve IBS-like symptoms.^98^

The Specific Carbohydrate Diet has been shown to improve symptoms and lead to mucosal healing in some pediatric patients with active CD.^99,100^ However, these were small and uncontrolled studies. A semivegetarian diet in Japan, compared to an omnivore diet, was associated with a significantly reduced relapse in patients with CD, though the study included only 22 patients who had achieved clinical remission either with infliximab or surgery.^101^

## UNANSWERED QUESTIONS

IBD has a relapsing and remitting course, with poor correlation between symptoms and pathologic findings. Meanwhile, diets are multifarious and inconsistent by nature, both day to day and person to person. These features make it difficult to study the role of nutrition in both the context of a hospitalization and longer time horizons. Furthermore, studies are challenged by obvious difficulty with blinding in randomized trials, the lack of a placebo control when studying whole food diets, bias resulting from misclassification of dietary intake, and frequent poor adherence to dietary recommendations.^97^

Specific to the hospital setting, there is a clear need for studies to establish the role of diet and nutrition among hospitalized patients with IBD, particularly those with fistula, abscess, severe acute colitis, and with anticipated nonurgent surgery but mild malnutrition. The role of NPO and clear liquid diet during active colitis needs better defined so as to reduce in-hospital undernutrition. We need more data regarding which patients gain benefit from a high-fiber diet and which disease conditions truly necessitate a low-fiber diet. We need randomized controlled trials to assess the appropriate dietary modifications for patients with fistula and/or abscess. New studies addressing diets for maintenance therapy could aid in recommendations on discharge.

Regarding diets for induction therapy, stronger evidence is needed to determine if EEN is comparable or even superior to corticosteroids for acute CD and if there is a role for PEN. A better understanding of the mechanisms of enteral nutrition may lead to the design of therapeutic diets, as either exclusive or adjunctive therapy. The role of diet in inducing and maintaining remission, and understanding the mechanisms of action of existing nutritional approaches is a research priority theme in the British Society of Gastroenterology guidelines.^5^ Meeting these research goals will be helpful for the care of patients with IBD both during and after hospitalization.

## CONCLUSIONS AND SUMMARY

Hereby, we offer a summary of the limited literature on nutritional considerations during hospitalization. Patients with IBD benefit from screening and nutritional support for malnutrition and nutritional deficiencies. Enteral nutrition and therapeutic diets appear promising as induction and maintenance therapies. There are no foods known to cause symptoms or mucosal damage for all patients, so patients are encouraged to isolate foods that may cause them symptoms. There are only a few reasons to enforce bowel rest or clear liquids for patients with IBD, such as bowel obstruction, uncontrolled sepsis, and the need for urgent or emergent surgery. Many patients, including those with penetrating or stricturing disease may benefit from feeding in whichever capacity tolerated, with enteral and PN used as needed to reach nutritional goals. As there remain gaps in our evidence base, we eagerly await more data on how diet can be used to optimize outcomes for patients with IBD.

## Data Availability

There were no primary data used in the preparation of this manuscript.

## Notes

Funding: T32-DK007740
